# Bibliometric analysis of knowledge maps and future trends of TIM-3 in cancer

**DOI:** 10.3389/fonc.2026.1766463

**Published:** 2026-02-10

**Authors:** Shuchi Lv, Shanchuan Huang, Langming Li, Kaifeng Lin, Hongliang Zhang, Yuqi Huang

**Affiliations:** 1 Department of Orthopaedics, Dongguan Humen Hospital, Dongguan, China; 2 Department of Breast Center, Dongguan Kanghua Hospital, Dongguan, China

**Keywords:** bibliometric, cancer, CiteSpace, immunotherapy, TIM-3, VOSviewer

## Abstract

**Objectives:**

This study conducted a bibliometric analysis of TIM-3 research in cancer in the past 25 years and expounded on the mainstream countries, institutions, authors, journals, and publications in this field. It intuitively depicts the evolution trajectory and future direction of TIM-3 research in the past 25 years.

**Materials and methods:**

The published literature on the role of TIM-3 in cancer research was retrieved from the WoSCC and PubMed databases. Various bibliometric tools (including VOSviewer and CiteSpace) were used to identify the historical features, the evolution of active topics, and emerging trends.

**Results:**

A total of 1466 articles were retrieved, with the main contributors including China, the United States, and Japan. The journal with the most published articles was Frontiers in Immunology. Anderson AC is the core author of this research field. The most common keyword is “TIM-3.” Furthermore, 37 clinical trial articles in the PubMed database were included. The analysis indicated that the main contributing countries were the United States, China, and Japan, with the United States being the pioneer of TIM-3 clinical research in cancer. Among these authors, Leena Gandhi published the highest number of articles. Keyword analysis indicated that most researchers experimentally validated and analyzed the characteristics of immune cells and biomarkers within the tumor microenvironment to predict clinical responses and mechanisms of resistance in cancer immunotherapy.

**Conclusion:**

The findings of this bibliometric study help researchers discover important topics and future research objectives by offering insights into the existing state and trends of TIM-3 in cancer.

## 1 Introduction

Cancer, the second leading cause of mortality worldwide, is a significant social, public health, and economic challenge in the 21st century (1). This highly complex disease is characterized not only by the unrestrained growth of malignant cells but also by alterations in immune responses (2). Currently, surgery, radiotherapy, chemotherapy, targeted therapy, and immunotherapy are the main treatment methods for cancer. With very few exceptions, the first four treatments act directly on the tumor itself (3, 4). But immunotherapy represents a unique approach to treating cancer in that it focuses on indirectly eliminating it by harnessing the power of the host immune system. The concept of cancer immunotherapy has been around for well over a century (5).

A central event in antitumor adaptive immunity is the activation of T cells. It has been discovered that T cell activation primarily occurs through a dual-signal pathway. First, it is activated when the T cell receptor (TCR) interacts with the antigen bound to the major histocompatibility complex (MHC) on the surface of the antigen-presenting cell (APC), secondly mediated by the binding of CD80 or CD86 on the surface of the APC to CD28 on the surface of the T cell (6). In the same year, Golstein et al. discovered a protein with a structure analogous to that of CD28, named Cytotoxic T Lymphocyte-Associated Antigen 4 (CTLA-4) (7–9). By late 1994, Allison et al. made a groundbreaking discovery that inhibiting CTLA-4 could enhance T cell antitumor activity and suppress tumor progression. The first demonstration that inhibiting a negative immune regulator could inhibit the progression of tumor growth (10), a methodology subsequently referred to as “Immune Checkpoint Inhibition (ICI)” by Allison (11). With this, a new era in cancer immunotherapy begins (12).

Currently, prominent immunosuppressive molecules (ICs) on T-cells include programmed cell death-1 (PD-1), CTLA-4, T-lymphocyte immunoglobulin and mucin domain-containing protein 3 (TIM-3), and lymphocyte activation gene-3 (LAG-3) (13). The methods via which the inhibition of immune checkpoint pathways enhances anti-cancer actions have been extensively researched, particularly with the CTLA-4 and PD-1 pathways (14, 15). In 2011, the U.S. Food and Drug Administration (FDA) sanctioned ipilimumab as the first immune checkpoint inhibitor for metastatic melanoma treatment (16). So far, numerous PD-1 inhibitor medicines have been marketed globally (17–19). Nonetheless, certain patients do not respond to these therapies or develop adaptive resistance. Consequently, it is essential to identify novel immune checkpoint molecules and surmount immune resistance. TIM-3 is one of the most promising immune checkpoint molecules among the many emerging ones (20).

TIM-3 (also known as CD366), initially identified in 2002, belongs to the TIM protein family of immunomodulatory proteins (21). The TIM family includes HAVCR1, HAVCR2, and TIMD4, which encode TIM-1, TIM-3, and TIM-4, respectively (22, 23). The genes that encode the TIM family are situated in a chromosomal area linked to allergies and asthma, and are T-cell surface inhibitory molecules (24). Among the TIM family members, TIM-3 has drawn the most attention because of its potential to exert both positive and negative effects on the regulation of primordial and adaptive immune reactions (25). TIM-3 is predominantly expressed in CD4+ TH1 helper T cells and CD8+ Tc1 cytotoxic T cells, and it is expressed in a subpopulation of Treg cells possessing enhanced suppressive capabilities (26). It is also expressed in monocytes and macrophages. Currently, TIM-3 ligands include galectin-9, phosphatidylserine (PtdSer), high-mobility group protein (HMGB1), and carcinoembryonic antigen cell adhesion molecule 1 (Ceacam1) (27). Numerous studies have demonstrated that Galectin-9 interacts with the TIM-3 carbohydrate chain, modulating Th1 cell immunity through the induction of apoptosis in Th1 cells (28), which might hinder anti-tumor immunity. Furthermore, TIM-3 can interact with another ligand, Ceacam1, in both cis and trans manners and is regarded as a potentially significant pathway for cancer immunotherapy (29). However, the interaction between the two in the tumor microenvironment is highly complex, and a unified conclusion has not been reached (30). Another reason for considering TIM3 as an attractive target for cancer immunotherapy is its co-expression with PD-1 (24). In preclinical colon cancer models, the combination of TIM-3 and PD-1 inhibitors has shown greater efficacy in tumor regression than monotherapy with either TIM-3 or PD-1 inhibitors (31, 32). TIM-3 also serves as a prognostic indicator for various cancers, such as colon cancer, gastric cancer, and non-small cell lung cancer, where high TIM-3 expression is associated with a negative prognosis for cancer survival (33). Moreover, evidence shows that elevated TIM-3 levels in circulating and tumor tissue cells of colorectal cancer patients are associated with an adverse disease course (34).

ICI has brought new hope for cancer treatment, but it still faces many challenges. At present, most cancer patients are not sensitive to ICI therapy, and even among the few patients who meet the treatment criteria, the occurrence of primary and acquired resistance results in a low treatment response rate. Primary resistance refers to the lack of clinical benefit after a certain course of treatment and evaluation, while acquired resistance refers to cancer that initially responds to immunotherapy but eventually recurs. The mechanisms of primary and acquired resistance are complex and diverse, involving multiple aspects such as intrinsic tumor factors, the tumor microenvironment, and individual patient differences (35, 36). In addition, biomarkers play a critical role, and accurate biomarkers are essential for predicting patient responses to ICI therapy, which helps optimize treatment outcomes and reduce adverse effects. However, reliable biomarkers are still lacking. Currently, FDA-approved biomarkers include PD-L1 expression, microsatellite instability-high (MSI-H), and tumor mutational burden (TMB), but there are limited standardized testing methods for their pan-cancer application, and their practical utility is constrained (37).

Bibliometrics aims to use multiple tools to collaboratively analyze existing literature databases and achieve multi-dimensional visualization. Unlike traditional reviews based on academic perspectives, it objectively presents the development trajectory of a field, constructs complex knowledge network maps, shows the overall picture of the research area, and provides a quantitative basis for research decision-making (38). This article aims to provide an overview of TIM-3 research in cancer through bibliometrics, highlighting influential contributors, institutions, and countries. It provides insights to guide future research. Moreover, by identifying the research hotspots in clinical trials, it can foster collaboration and stimulate innovation, exploring new clinical directions in cancer immunotherapy.

## 2 Materials and methods

Bibliometrics was initially proposed in the early 20th century, but it gained significant attention only in 1969, when it emerged as a distinct research discipline. Bibliometrics is a quantitative method used to examine existing research in specific fields and periods (39). This not only enables the study of the history of scientific research in a particular domain, providing an evidence-based assessment of scientific productivity, but also aids in identifying potential future research trends, emerging hotspots, and collaboration patterns. In addition, it offers crucial information for research institutions in strategic planning and resource allocation (40). Currently, it is widely employed in analyzing the literature (41). The analytical process yielded detailed information about authors, countries, institutions, keywords, journals, and references. Modern computer technology is utilized to explore the inherent relationships among these data, and graphical and visual outputs complement the literature analysis (42). With the continuous growth in the number of publications and the increasing significance of research impact, bibliometrics will remain crucial in assessing research.

In this study, software tools including CiteSpace (version 6.4.R1) and VOSviewer (version 1.6.20) were employed for data extraction and analysis, ultimately constructing a knowledge map. Microsoft Office Excel 2021 was used to process the data and generate graphs representing the annual publication trends. Additionally, Pajek (version 5.17) and Scimago Graphic (version 1.0.46) were employed for further processing of the knowledge map produced by VOSviewer. These software programs offer unique advantages and can effectively complement each other. VOSviewer is a powerful tool for co-authorship and co-occurrence analysis, featuring an embedded clustering algorithm that produces various visualization views of the literature, including network, overlay, and density visualization, all characterized by simplicity and aesthetically pleasing (43). This study utilized VOSviewer to create visualization views of keywords, authors, institutions, and other aspects of the literature. CiteSpace, a tool for citation analysis and research field visualization, facilitates the depiction of structures, layouts, and prospective research trajectories inside an academic field (44, 45). In this context, CiteSpace was applied to generate dual map overlays of journals and conduct burst analyses of keywords and co-cited literature. The time periods span from 2003 to 2025, with each time slice being one year. The alluvial flow diagram was designed to illustrate evolving network flow patterns over time, named after alluvial fans formed by the natural deposition of sediment carried by flowing water (46). The division and merging of thematic patterns can be viewed as multiple streams that flow smoothly over time. To generate an alluvial map, CiteSpace was used to produce a series of individual networks for the co-occurring keywords. These networks were then loaded into an alluvial generator to create an alluvial flow diagram(http://www.mapequation.org/apps/AlluvialGenerator.html).

This study utilizes the Web of Science Core Collection (WoSCC) as the data source. Additionally, we supplement the clinical research trends in this field through PubMed searches to ensure the comprehensiveness of the data and the rigor of the methodology. WoSCC is a high-quality digital literature resource and one of the most extensively utilized databases currently available. Compared to other databases, such as Scopus and Medline, it is considered the most appropriate for bibliometric studies (38, 47, 48).

**Figure 1:**
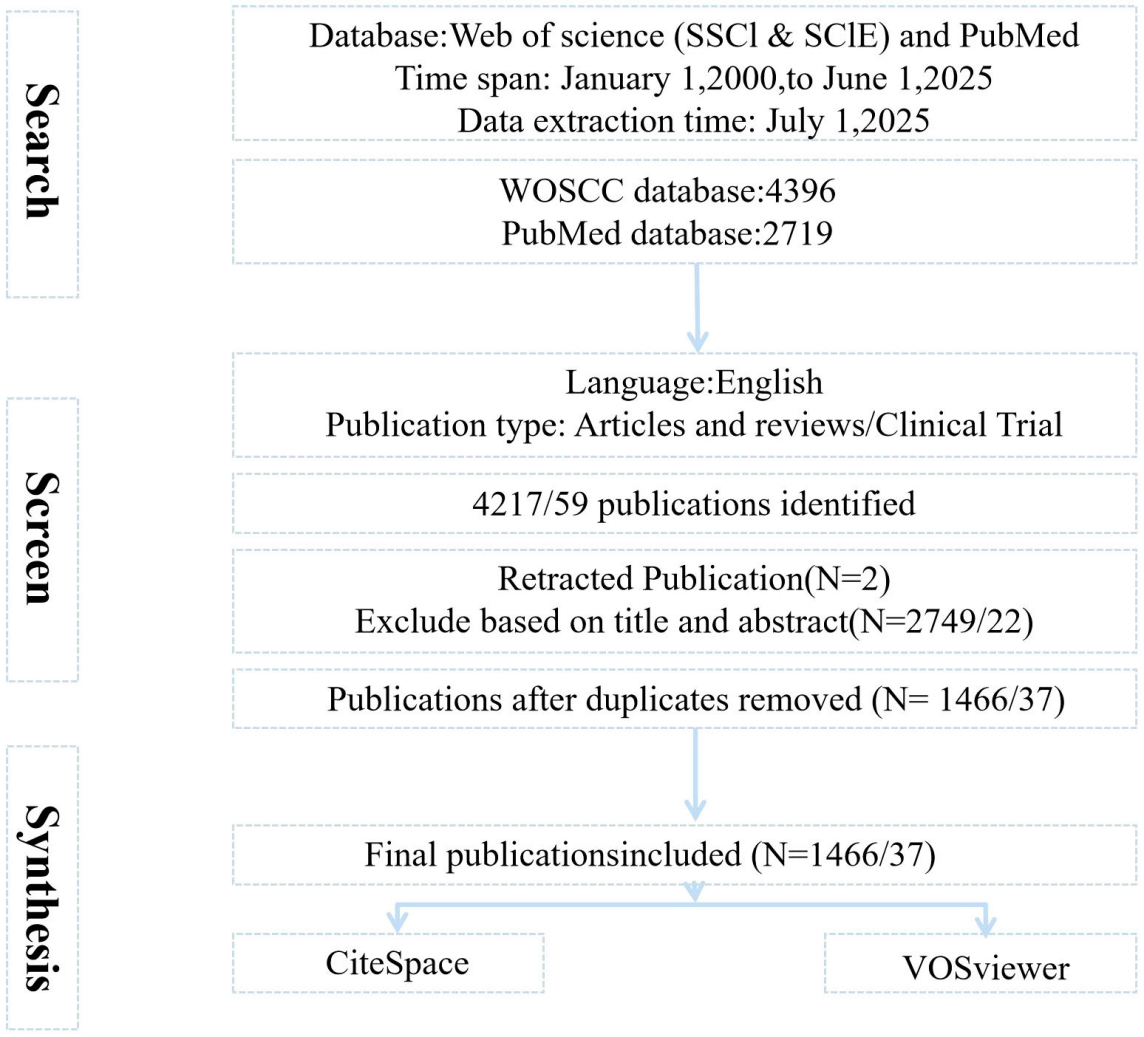
Flowchart of the literature searching and screening.

For a thorough and precise analysis of the gathered information, the Social Science Citation Index (SSCI) and the Science Citation Index Expanded (SCIE) were chosen as primary indices. The search strategy employed was TS = (“T-Cell Immunoglobulin And Mucin Domain 3” OR “T Cell Immunoglobulin Mucin 3” OR “T-Cell Immunoglobulin And Mucin Domain-Containing Protein 3” OR “T-Cell Immunoglobulin Mucin Family Member 3”) OR TS= (Tim-3 OR TIM3 OR Timd3 OR “HAVCR2” OR “FLJ14428”) OR TS= (CD366 OR CD366 Antigen*) AND TS= (cancer* OR tumor* OR Neoplasia*). The PubMed search strategy was (cancer* OR tumor* OR neoplasia* [MeSH Terms]) AND (T-Cell Immunoglobulin And Mucin Domain 3 OR T Cell Immunoglobulin Mucin 3 OR T-Cell Immunoglobulin And Mucin Domain-Containing Protein 3 OR T-Cell Immunoglobulin Mucin Family Member 3 OR Tim-3 OR TIM3 OR Tim-3 OR “HAVCR2” OR “FLJ14428” [MeSH Terms]), with a time from January 2000 to June 2025. The search time was up to June 1, 2025. All searches were completed on July 1, 2025, to minimize any potential biases arising from database updates. Inclusion criteria (1): The type of literature is research articles and reviews (including meta-analyses) (2); Language is English (3); Research subjects: primarily focused on the study of TIM-3 in cancer, covering its mechanisms of action, therapeutic applications, etc. Exclusion criteria (1): The type of literature is conference papers, theses, duplicate publications, or news (2); the literature content is unrelated to the topic or lacks information, such as authors, author affiliations, or publication year. Consequently, 1466 articles were obtained from the WoSCC database, and 37 clinical trial articles were obtained from the PubMed database, downloaded in plain text and tab-delimited file formats (**Figure 1**).

## 3 Results

### 3.1 Data collection

**Figure 2:**
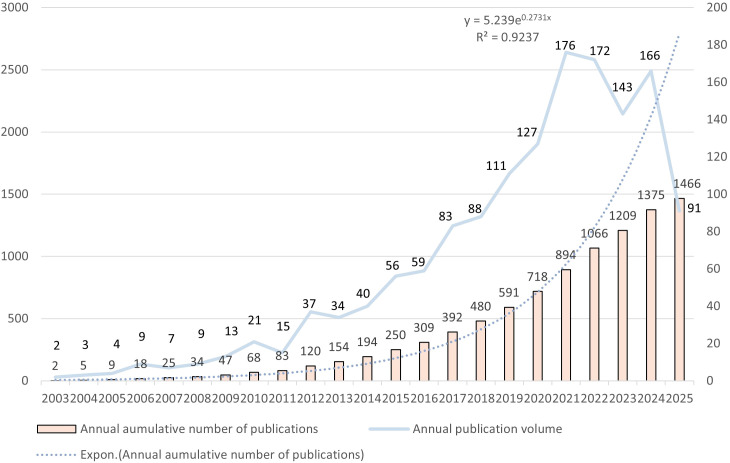
A map of the annual and cumulative publications time trend.

The 1,466 articles used in this study were authored by 10,079 authors from 1,767 organizations across 67 countries and published in 3,446 journals, citing 44,727 articles from 3,797 journals. **Figure 2** illustrates the annual and cumulative publication numbers associated with TIM-3 in the field of cancer from 2000 to 2025. The first study on TIM-3 in cancer was published in 2003, there were fewer than 5 publications per year until 2005. From 9 articles in 2005 to 83 articles in 2011, the number of cumulative publications steadily increased but remained below 100. In the subsequent phase, following the advent of ICIs, the cumulative number of publications increased rapidly, particularly after 2020. Additionally, we evaluated total publications and the year of publication using an exponential growth function, finding a strong correlation that matched the trend in cumulative publication quantity (R^2^ = 0.9237). This robust link signifies that TIM-3 has garnered heightened interest from researchers in the field of cancer and is a focal point in cancer research.

### 3.2 Bibliometric analysis of authors and co-cited authors

Analyzing the authors of the literature provides a more profound understanding of the leading scholars and research trends in the field. A total of 10,079 individuals authored 1,466 articles. **Table 1** presents an extensive overview of the top 10 authors, including their respective names, publications, and average citations per publication. The top 10 authors collectively published 172 articles, accounting for approximately 11.73% of the total literature. Kuchroo VK, stands out as the most prolific author with 46 articles, representing 3.14% of the total. Anderson AC (with 21 publications and 7,228 citations), and Hirashima Mitsuomi (with 19 publications and 3,433 citations), follow closely. Further analysis reveals that among the top 10 authors, most are from the United States and China.

**Table 1:** Top 10 productive authors in the field of cancer research pertaining to TIM-3.

Rank	Author	Documents (n%)	Citations	Average citation/publication
1	Kuchroo VK (USA)	46 (3.14%)	14059	305.63
2	Anderson AC (USA)	21 (1.43%)	7228	344.19
3	Hirashima Mitsuomi (Japan)	19 (1.30%)	3433	180.68
4	Kane LP (USA)	14 (0.95%)	712	50.86
5	Akiba Hisaya (Japan)	12 (0.82%)	2114	176.17
6	Elkord E (Oman)	12 (0.82%)	629	52.42
7	Gencheng Han (China)	12 (0.82%)	530	44.17
8	Yangqiu Li (China)	12 (0.82%)	302	25.17
9	Beifen Shen (China)	12 (0.82%)	530	44.17
10	Yagita Hideo (Japan)	12 (0.82%)	3019	251.58

**Figure 3:**
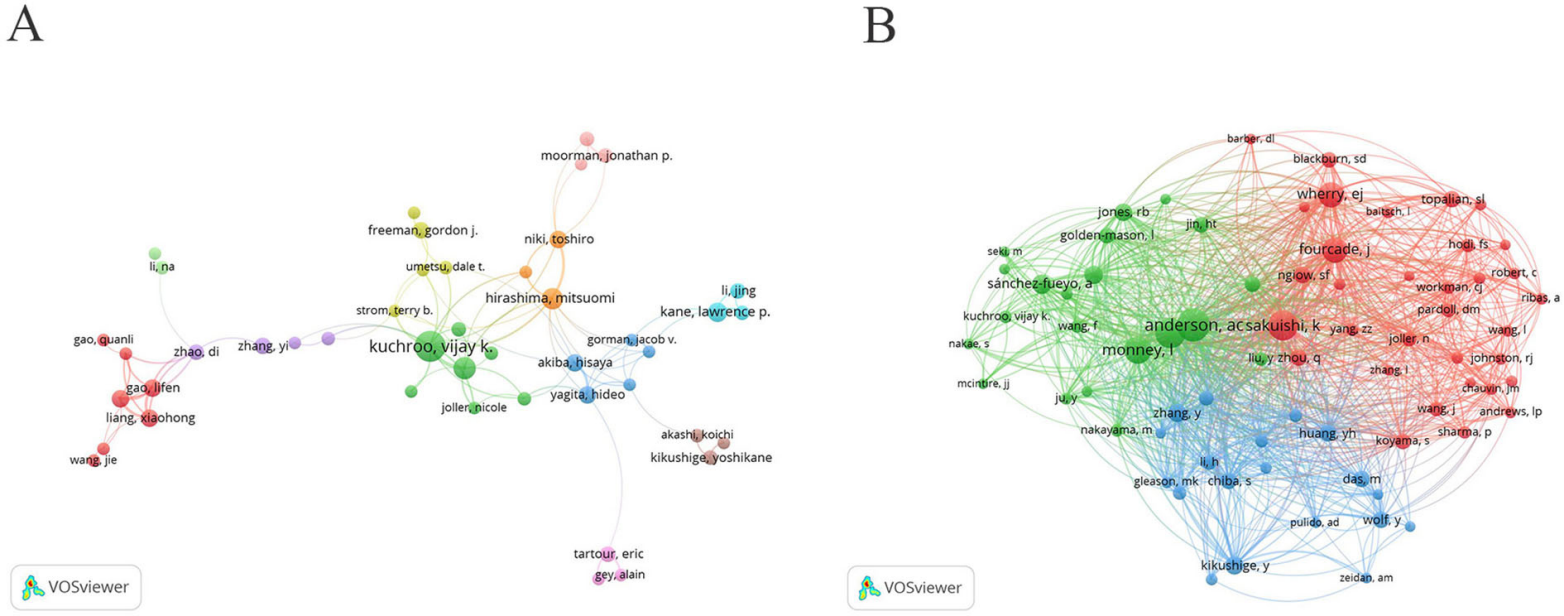
**(A)** The authors collaboration network (Top101); **(B)** The co-cited authors collaboration network (Top73).

After setting a minimum publication threshold of 5 articles and using VOSviewer to merge synonyms for authors, the names “Kuchroo, Vijay,” “Kuchroo, VK,” and “Kuchroo, Vijay K” were combined into “Kuchroo, Vijay K.” A visual analysis was conducted on the top 101 authors, resulting in a collaborative network diagram describing them (**Figure 3A**). We find that the top three authors with the most publications, Kuchroo VK, Anderson AC, and Hirashima Mitsuomi, are primarily located in the green and orange clusters. The largest cluster, represented in red, consisted of 7 authors who show strong potential as an emerging group, with a total of 53 publications (3.61%). Additionally, we used VOSviewer for the co-citation analysis. By setting a minimum citation threshold of 70, we generated an interconnected author network diagram consisting of 73 authors to illustrate their influence on the field of TIM-3 research in cancer (**Figure 3B**). Among the top 10 co-cited authors (**Table 2**), Anderson AC, ranks first with 536 citations, followed by Kaori Sakuishi (466 citations) and C Zhu (450 citations). It’s worth noting that Anderson AC, is one of the top three authors in terms of publication volume in this research field.

**Table 2:** Top 10 productive co-citation authors in the field of cancer research pertaining to TIM-3.

Rank	Co-cited author	Country	Citations
1	Anderson AC	USA	536
2	Kaori Sakuishi	Japan	466
3	C Zhu	USA	450
4	Monney L	USA	385
5	Fourcade J	USA	342
6	Wherry EJ	USA	330
7	Sánchez-Fueyo A	United Kingdom	204
8	Sabatos CA	USA	196
9	Y Zhang	China	193
10	Kikushige Y	Japan	188

### 3.3 Bibliometric analysis of journals and co-cited journals

**Figure 4:**
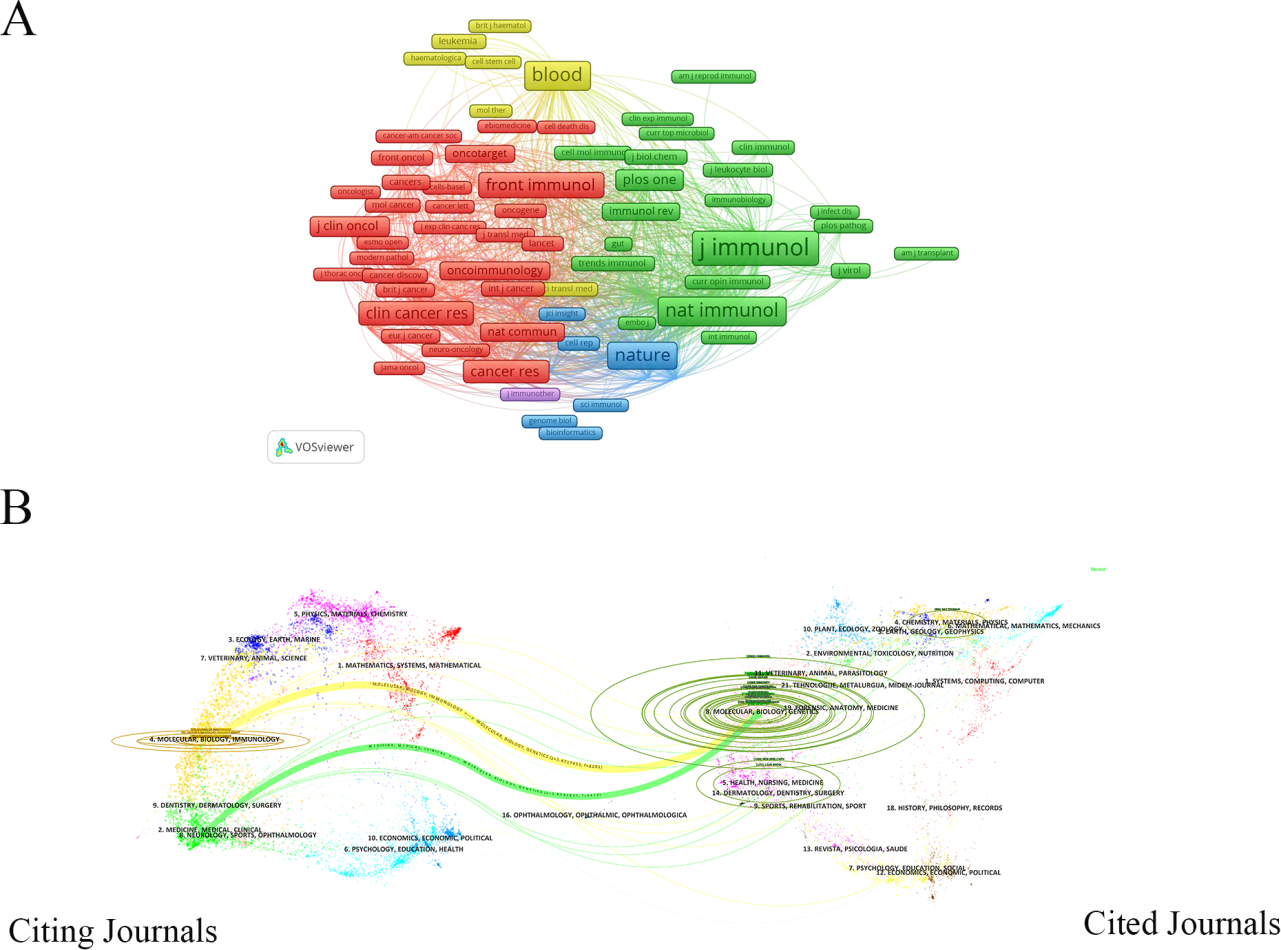
**(A)** The co-citation of cited journals network; **(B)** The dual-map overlay of journals related to TIM-3 research in the cancer field.

Analyzing literature journals to identify authoritative ones can facilitate researchers’ understanding of current trends and effective tracking of research hotspots in their fields. A total of 346 journals have published literature on TIM-3 research in cancer. **Table 3** lists the top 10 journals with the largest publication volumes. Among these, Frontiers in Immunology led to 133 publications, followed by the Journal of Immunology (57 publications) and Cancers (47 publications), all over 40. This result indicates that these journals have a significant influence on TIM-3 research. 60% of the top 10 journals originated from the United States, 30% from Switzerland, and the remaining 10% from Greece. It’s worth noting that Oncotarget has been deselected for coverage on Web of Science. The Journal of Immunology has the highest citation count (4,008 citations), which is significantly higher than that of the other journals. Based on the average citation count per publication, Clinical Cancer Research ranks first with an average of 86.45 citations. The second and third places are occupied by the Journal of Immunology (average 70.32 citations) and Oncoimmunology (average 557.75 citations), respectively. **Figure 4A** displays a network visualization of the most commonly co-cited journals. The top three journals with the largest nodes are the Journal of Immunology (3,566 citations), Nature Immunology (2,476 citations), and Blood (2,475 citations). Most of the top 10 journals by publication volume were related to immunology, while the 10 highly cited journals were mostly associated with cancer, which aligns with the dual-map overlay analysis. The dual-map overlay of journals in **Figure 4B** illustrates the subject distribution of the academic journals (49). The citing journals are positioned on the left side of the map, and the cited ones are on the right. These labels represent the disciplines covered by journals. From left to right, the colored lines depict the reference paths. There are two distinct citation paths: the yellow citation path indicates that Molecular, Biology, and Genetic journals are cited by Molecular, Biology, and Immunology journals, and the green citation path suggests that research and applications in Molecular, Biology, and Genetics are frequently cited by Medicine, Medical, and Clinical journals.

**Table 3:** Top 10 journals in the field of cancer research pertaining to TIM-3.

Rank	Source	Documents	Citations	Average citation/publication
1	Frontiers in Immunology(Switzerland)	133	3382	25.43
2	Journal of Immunology(USA)	57	4008	70.32
3	Cancers(Switzerland)	47	525	11.17
4	cancer immunology immunotherapy(USA)	41	990	24.15
5	frontiers in oncology(Switzerland)	40	785	19.63
6	journal for immunotherapy of cancer(USA)	40	1360	34.00
7	oncoimmunology (USA)	36	2079	57.75
8	clinical cancer research(USA)	22	1902	86.45
9	Oncology Letters(Greece)	22	372	16.91
10	oncotarget(USA)	21	783	37.29

### 3.4 Bibliometric analysis of publications by country and institutions

#### 3.4.1 Country analysis

To understand which countries have the most substantial contributions to TIM-3 research on cancer, we conducted an analysis of publication volume by country. It is important to note that publications from certain countries were merged. Due to synonymy, such as those from Taiwan, which were combined with publications from China. Similarly, publications from England, Scotland, Northern Ireland, and Wales were consolidated under the United Kingdom. **Table 4** shows the top 10 countries by publication quantity, along with their citation frequencies and average number of citations per publication. The analysis revealed that China leads the pack with 622 publications between 2000 and June 2025, followed by the United States (410 publications) and Japan (130 publications), and all other countries have fewer than 100 publications. It is noteworthy that the United States has a total of 36,466 citations, nearly double the 16,101 citations of China in second place, while Japan ranks third with a total of 8,573 citations. Further analysis of the average number of citations per publication shows that the United States tops the list with an average of 88.94 citations, followed by Japan (65.95 citations) and France (63.83 citations). The United States achieved high rankings in both publication volume and average citations per publication, reflecting its substantial impact in this field. The country holds a leading position in terms of both quantity and quality of published articles.

**Table 4:** Top 10 countries in the field of cancer research pertaining to TIM-3.

Rank	Country	Documents(n%)	Citations	Average citation/publication
1	China	622 (42.43%)	16101	25.89
2	USA	410 (27.97%)	36466	88.94
3	Japan	130 (8.87%)	8573	65.95
4	Germany	89 (6.07%)	3907	43.90
5	United Kingdom	67 (4.57%)	3446	51.43
6	France	60 (4.09%)	3830	63.83
7	Canada	53 (3.62%)	3053	57.60
8	Italy	49 (3.34%)	1853	37.82
9	Australia	36 (2.66%)	2234	57.28
10	Iran	36 (2.66%)	537	13.77

**Figure 5:**
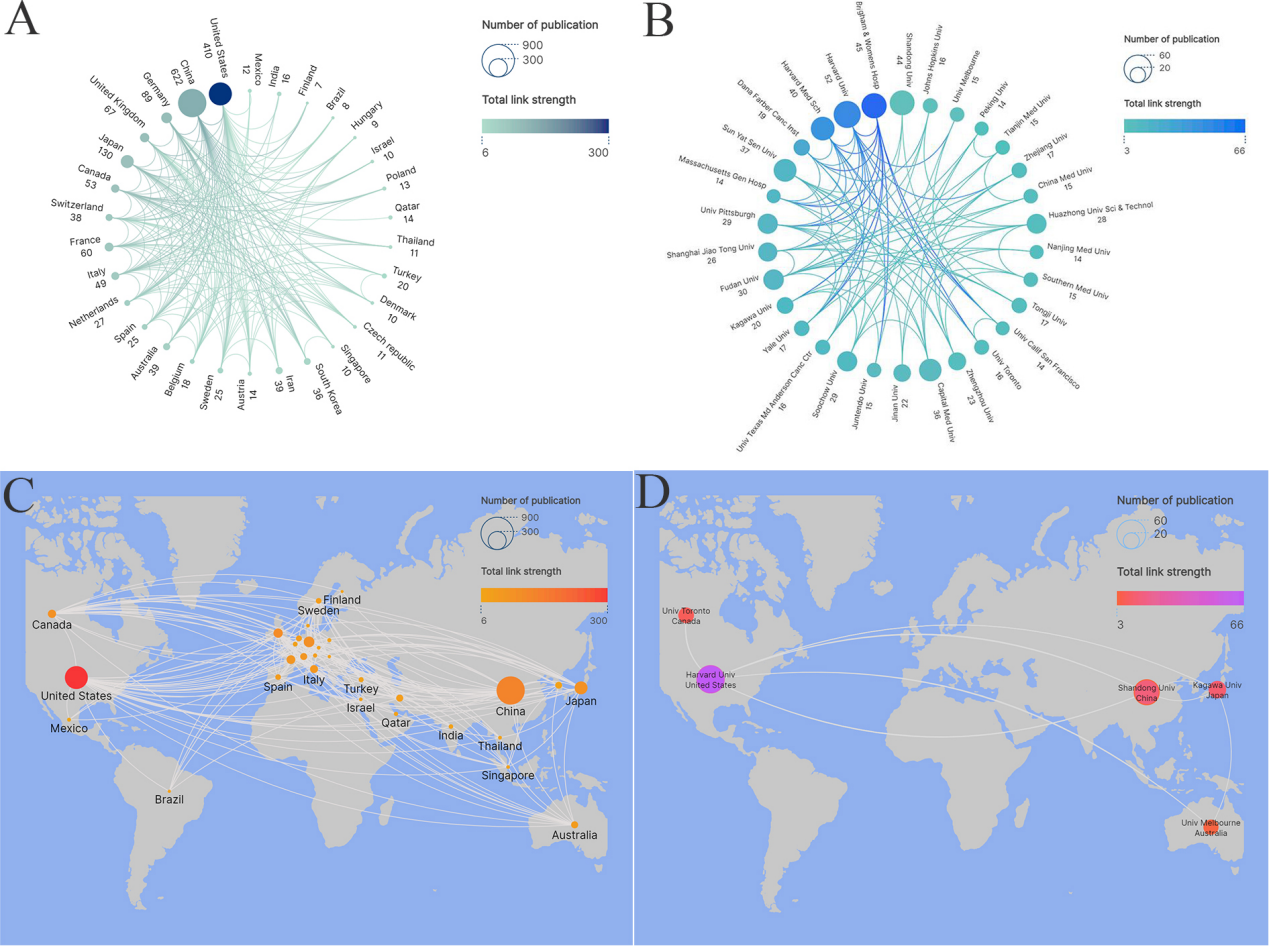
Top 30 countries **(A)** and organizations **(B)** network for TIM-3 research in cancer. The top 30 countries **(C)** and organizations **(D)** on the world map for TIM-3 research in cancer.

Furthermore, we conducted a visual analysis of publication volume and collaborative partnerships among the top 30 countries using VOSviewer and Scimago Graphic (**Figure 5A**). The centrality index was employed to measure the significance of the network nodes (50). Our findings revealed that while China had the highest publication volume, the centrality analysis indicated that the United States (0.96) played a central role in the collaboration network and maintained close ties with the majority of countries. Additionally, we visualized the 30 countries on a global map (**Figure 5C**), mostly in Europe. It is worth mentioning that the countries with the highest number of publications were primarily located in North America (mainly the United States) and Asia (mainly China).

#### 3.4.2 Institutional analysis

To understand which institutions make the most substantial contributions to TIM-3 research in cancer, we conducted an analysis focusing on the publication output of various institutions. **Table 5** presents data on the top 10 institutions by publication quantity, along with their citation frequencies and average number of citations per publication. Our analysis revealed that the leading institutions primarily originated from the United States and China and authored 370 articles, accounting for 25.24% of the total publications. Harvard University tops the list with 52 publications (representing 3.55% of the total), earning recognition as a significantly influential institution in terms of its publication output. Brigham & Women’s Hospital (with 45 publications, accounting for 3.07% of the total) and Shandong University (with 44 publications, accounting for 3% of the total) occupied second and third positions, respectively. Furthermore, using VOSviewer and Scimago Graphica, we conducted a visual analysis of the publication volume and partnership networks of the top 30 institutions (**Figure 5B**). Our data analysis indicated that most nodes have low centrality scores, indicating that the majority of institutions still have limited influence, and there is inadequate collaboration among them. Additionally, we visualized these 30 institutions on a global map (**Figure 5D**), revealing that most were located in North America (primarily the United States) and Asia (mainly China). It’s worth mentioning that Australia has only one institution: the University of Melbourne.

**Table 5:** Top 10 organizations in the field of cancer research pertaining to TIM-3.

Rank	Organization	Documents(n%)	Citations	Average citation/publication
1	Harvard Univ (USA)	52 (3.55%)	14699	282.67
2	Brigham & Womens Hosp (USA)	45 (3.07%)	10246	227.69
3	Shandong Univ (China)	44 (3.00%)	1278	29.05
4	Harvard Med Sch (USA)	40 (2.73%)	3546	88.65
5	Sun Yat-sen University (China)	37 (2.52%)	512	13.84
6	Capital Med Univ (China)	36 (2.46%)	1107	30.75
7	Fudan Univ (China)	30 (2.05%)	1075	35.83
8	Soochow Univ (China)	29 (1.98%)	640	22.07
9	Univ Pittsburgh (USA)	29 (1.98%)	3013	103.90
10	Huazhong Univ Sci & Technol (China)	28 (1.91%)	1973	70.46

### 3.5 Co-cited references and references with citation bursts

Using co-citation analysis, this study examined the literature related to TIM-3 research in cancer from the past two decades, both domestically and internationally. This study was designed to examine the developmental trends and prospects of TIM-3 research in cancer. The theory of document co-citation, which originated in 1973 by Henry Small and Irina Mashakova, is still a prevalent method in scientific quantitative research (38, 51, 52). This method analyzes citation relationships between publications, enabling the retrieval of key documents that significantly influence the research field (53).

**Table 6** presents a summarized list of 10 co-cited references regarding TIM-3 research in cancer, with citation counts ranging from 153 to 385. The article titled “Th1-specific cell surface protein TIM-3 regulates macrophage activation and severity of an autoimmune disease” by Laurent Monney, published in Nature, received the highest number of citations (385) (21). The second and third most cited articles were “The TIM-3 ligand Galectin-9 negatively regulates T helper type 1 immunity” (365 citations) and “Targeting TIM-3 and PD-1 pathways to reverse T cell exhaustion and restore anti-tumor immunity” (343 citations), respectively. All three references were published in journals classified as Q1 (28, 31). Additionally, half of the references were cited at least 200 times, and almost all the studies were published before 2010, except for the 9th and 10th references.

**Table 6:** Top 10 co-cited references in the field of cancer research on TIM-3.

Rank	Co-cited reference	Country	Co-citation	IF (2025)
1	Monney L, 2002, nature, v415, p536	USA	385	42.78
2	C Zhu, 2005, nat immunol, v6, p1245	USA	365	20.48
3	Sakuishi K, 2010, j exp med, v207, p2187	USA	343	11.74
4	Fourcade J, 2010, j exp med, v207, p2175	USA	238	11.74
5	Sánchez-Fueyo A, 2003, nat immunol, v4, p1093	USA	202	20.48
6	Sabatos CA, 2003, nat immunol, v4, p1102	USA	196	20.48
7	Anderson AC, 2007, science, v318, p1141	USA	186	41.85
8	Jones RB, 2008, j exp med, v205, p2763	Canada	169	11.74
9	Anderson AC, 2016, immunity, v44, p989	USA	167	22.56
10	Chiba S, 2012, nat immunol, v13, p832	Japan	153	20.48

**Figure 6:**
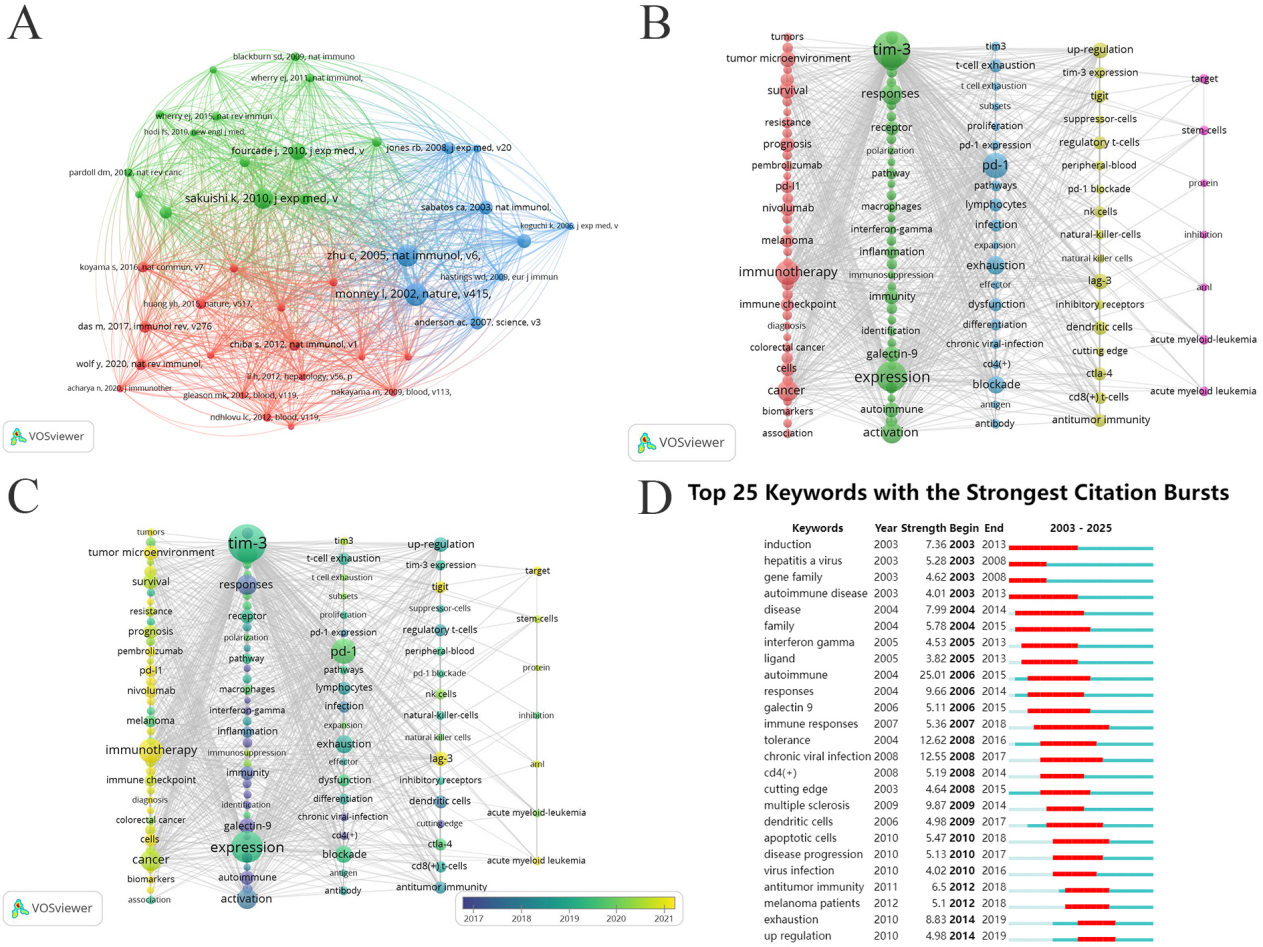
**(A)** The co-citation of cited references network; **(B)** The frequency keywords network (Top 118); **(C)** Dynamics and trends of the frequency keywords over time (Top 118); **(D)** Top 25 keywords with citation bursts.

To further explore the citation structure of TIM-3 research in cancer, we employed VOSviewer to generate a co-citation map. The minimum citation threshold for a cited reference was set at 70, and 40 articles were analyzed. The resulting co-citation relationships are shown in **Figure 6A**. Notably, highly co-cited literature can be categorized into three major clusters, represented by three distinct colors in the figure. The green cluster focuses primarily on targeting the TIM-3 pathway to elicit anti-tumor immunity, with the seminal work by Kaori Sakuishi et al., “Targeting TIM-3 and PD-1 pathways to reverse T cell exhaustion and restore anti-tumor immunity,” leading the pack (31). Most articles in this cluster were published before 2015. The red cluster delves deeper into the role of TIM-3 in modulating autoimmune and anti-tumor immune responses, with publications spanning from 2000 to 2020. The blue cluster centers around TIM-3 role in regulating immune cell function, led by Laurent Monney’s “Th1-specific cell surface protein Tim-3, which regulates macrophage activation and severity of an autoimmune disease” (21). The majority of the articles in this cluster were published before 2010.

Additionally, we utilized CiteSpace to generate an emergence map of references that saw a citation spike within a brief timeframe across certain years, allowing us to observe the developing frontier fields. **Supplementary Figure S1** (Supplementary Material) presents the top 25 highly cited references with citation bursts. The first citation surge appeared in 2003 (21). The article that first exhibited attracted significant interest upon publication, with a burst strength of 33.45, and the burst duration lasted for 10 years, from 2003 to 2010. Monney et al. experimentally demonstrated that TIM-3 antibody binding to the TIM-3 receptor can activate macrophages and enhance their proliferation, indicating that TIM-3 negatively affects macrophage activity. An article by Kuchroo VK exhibited a burst strength of 13.46 from 2003 to 2011 (23). This article primarily describes various TIM family members in mice and humans and discusses the genetic and functional evidence of their roles in regulating autoimmune and allergic diseases. The third burst period, which occurred from 2004 to 2011, featured Alberto Sánchez-Fueyo as the lead author and demonstrated a burst strength of 33.67 (54). They found that blockade of the TIM-3 pathway accelerated diabetes in non-obese diabetic mice and prevented the acquisition of transplantation tolerance induced by costimulatory blockade. These effects were mediated, at least in part, through inhibition of the antigen-specific immunosuppressive function of the CD4^+^CD25^+^ regulatory T cell population.

### 3.6 Keyword analysis

Keywords represent the core and essence of an article. Through co-occurrence analysis of these keywords, it is possible to identify cutting-edge developments within specific research fields (55). There exist 4,441 keywords throughout 1,466 publications. Utilizing VOSviewer, a keyword co-occurrence network view was constructed for these 1,466 publications. Visualization focuses on 118 keywords with a frequency of 22 or higher, and the network diagram was optimized using Pajek. Larger circular nodes indicate a higher frequency of keyword appearance, signifying a greater representation of research hotspots within the field. The lines linking the nodes signify the association strength, where thicker lines denote more co-occurrences within the same literature. Node colors represent different clusters, and it is noteworthy that “TIM-3,” “expression,” “cancer,” “PD-1,” and “immunotherapy” emerge as the four most significant nodes within the network. Additionally, **Figure 6B** illustrates an overlay visualization that shows the top 118 keywords from 2000 to 2025. Five clusters were identified. The green cluster comprises 36 keywords primarily related to TIM-3, T-cells, response, receptor, pathway, expression, activation, and immunity. The red cluster contains 38 keywords, predominantly associated with survival, PD-L1, immunotherapy, and cancer. The blue cluster encompasses 20 keywords, primarily linked to prominent terms such as PD-1, LAG-3, exhaustion, dysfunction, and blockade. The yellow cluster included 17 keywords, such as LAG-3, up-regulation, and antitumor immunity. Finally, the purple cluster consisted of 7 keywords primarily connected to inhibition, target, and stem-cells.

Additionally, **Figure 6C** shows that prominent nodes, including expression, TIM-3, and PD-1, mainly appeared in 2019. Following 2021, other significant keywords such as “immunotherapy,” “prognosis,” and “cancer” began to appear. By employing CiteSpace for citation burst analysis, the top 25 keywords with the strongest citation bursts were arranged chronologically from top to bottom, revealing the evolution of TIM-3 in cancer research from 2000 to 2025. As shown in **Figure 6D**, it can be seen that the first keywords with strong citation rates in this field originated in 2003, including induction, hepatitis a virus, gene family, and autoimmune disease. Among these keywords, only “autoimmune” exhibited a burst strength exceeding 20. In addition, we specifically focused on all 15 burst keywords from the burst period to 2025, as they may represent future research hotspots in this field. For instance, the keyword “immune checkpoint inhibitors” had a burst strength of 7.97 from 2021 to 2025, and the keyword “tumor microenvironment” exhibited a burst strength of 8.09 from 2023 to 2025 (**Supplementary Material Table S1**).

**Figure 7:**
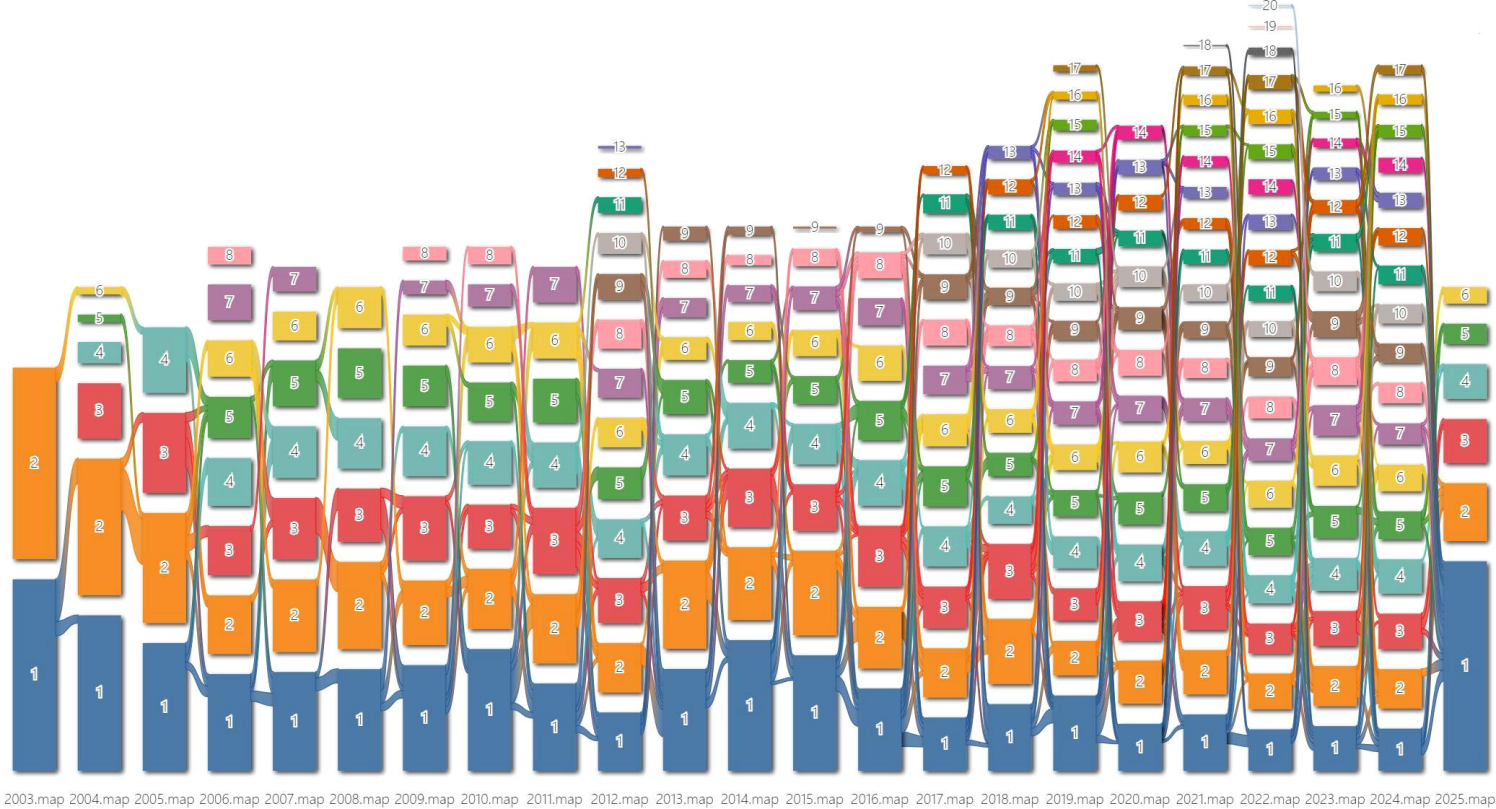
Keyword alluvial map from 2003 to 2025. X axis: Time slice. Y axis: Counting of modules. Number: Order of modules on each time slice, sorted by the number of nodes.

**Table 7** lists the top 20 most frequently occurring keywords. These primarily consist of terms related to cancer development, progression, and survival prognosis, such as exhaustion, survival, and prognosis. Additionally, the tumor microenvironment and immune checkpoint-related terms, including PD-1, blockade, TIM-3, and LAG-3, are prominent. It is noteworthy that activation, responses, and T-cells also exhibit significant attention, indicating that increasing numbers of researchers are deepening their investigations. The study of the TIM-3 immune checkpoint has garnered broader interest. As illustrated in **Figure 7**, connected keywords can be grouped into specific research clusters. When keywords are recombined, these clusters diverge or converge to form new modules. The alluvial map traces the evolving landscape of research from 2003 to 2025, highlighting the emergence and interconnections between various themes over time. The top five keywords with the highest flow in each cluster per year are listed in **Supplementary Table S2** (Supplementary Material).

**Table 7:** Top 20 keywords in terms of frequency in the field of cancer research on TIM-3.

Rank	Keyword	Occurrences	Rank	Keyword	Occurrences
1	TIM-3	675	11	Survival	115
2	Expression	454	12	Galectin-9	109
3	PD-1	294	13	Lag-3	101
4	Immunotherapy	267	14	Up-Regulation	101
5	Cancer	205	15	Tumor Microenvironment	99
6	Activation	193	16	Autoimmune	93
7	Responses	190	17	Prognosis	91
8	T-Cells	177	18	Immunity	90
9	Exhaustion	142	19	Receptor	84
10	Blockade	131	20	Cells	77

### 3.7 Analysis of the PubMed database

#### 3.7.1 Analysis of author and country co-occurrence

**Figure 8:**
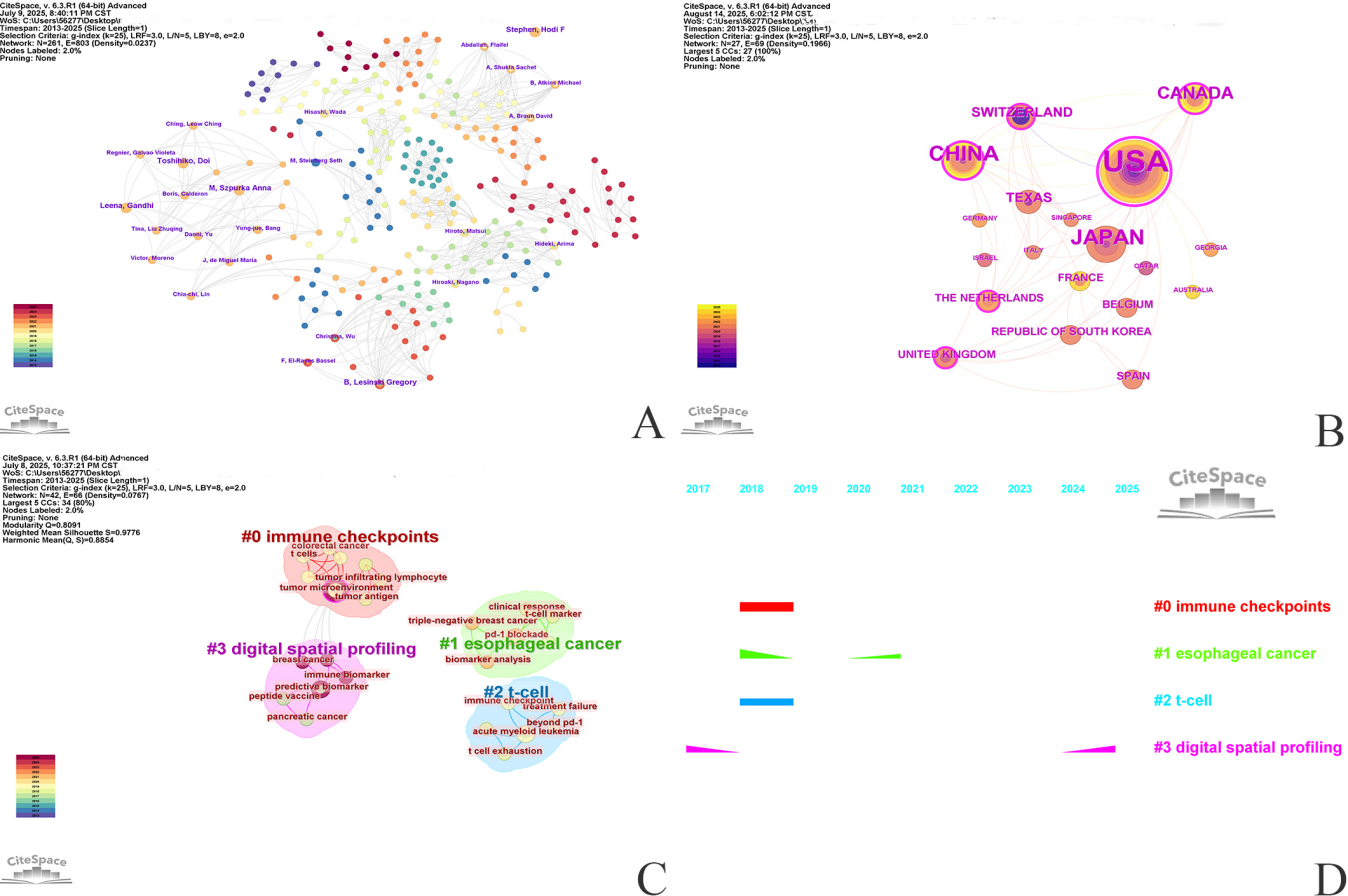
Source from the PubMed database, co-occurrence analysis of authors related to TIM-3 cancer clinical trials **(A)**, co-occurrence analysis by country **(B)**, keyword clustering analysis **(C)**, and temporal dimension of keyword clustering **(D)**.

A co-occurrence analysis of the authors (**Figure 8A**) and countries was performed using CiteSpace. The 37 publications were co-authored by 258 authors from 19 countries. **Supplementary Table S3** (Supplementary Materials) lists the top 10 most prolific authors along with their names, number of publications, and affiliated countries. Leena Gandhi was the most prolific author, having published 3 articles, accounting for 8.11% of the total literature. She was followed by M. Szpurka, Anna, and Stephen, Hodi F (each with 3 publications). All three top authors were affiliated with institutions in the United States. Further analysis revealed that 9 out of the top 10 authors were from the United States, while one was from Japan. Similarly, a co-occurrence analysis of the countries was performed using CiteSpace (**Figure 8B**). The synonyms of some countries, such as publications from Taiwan, were merged with those of China. Likewise, publications from England, Scotland, Northern Ireland, and Wales were merged into those of the United Kingdom. 19 countries contributed to clinical trials in the field of TIM-3 cancer research. A large number of nodes and abundant connections indicate strong scientific collaboration among countries. The top three countries by node size were the United States, China, and Japan. Among these, the United States exhibited the most extensive linkages, maintaining close ties with countries such as Canada, Switzerland, and Japan. This finding is consistent with the results of the WOS database country analysis, underscoring the prominent role of the United States in the collaborative network and highlighting its leading position in clinical trial research related to TIM-3 in cancer.

#### 3.7.2 Keyword clustering analysis

Keywords exhibit strong intrinsic correlations and can form distinct clusters based on their affinity. An in-depth analysis of these keyword clusters offers information about the evolving subdomains within TIM-3 and cancer clinical research. The keyword clusters are shown in **Figure 8C**. They can be categorized into four groups: #0 immune checkpoint (primarily including tumor microenvironment, T regulatory cells, colorectal cancer, T cells, infantile fibrosarcoma, and tumor-infiltrating lymphocyte), #1 esophageal cancer(primarily including PD-1 blockade, esophageal cancer, T-cell marker, clinical response, biomarker analysis, and triple-negative breast cancer), #2 T-cell (primarily including acute myeloid leukemia, immune checkpoint, treatment failure, beyond PD-1, NK cells, and T cell exhaustion), and #3 digital spatial profiling (primarily including predictive biomarker, digital spatial profiling, breast cancer, immune biomarkers, pancreatic cancer, and peptide vaccine).

As shown in **Figure 8D**, the temporal dimension is predominantly concentrated after 2017. Cluster #0 (immune checkpoint) and cluster #2 (T-cell) began to emerge in 2018. Cluster #3 (digital spatial profiling), which involves the analysis, interpretation, and processing of biomedical signals using signal processing techniques, was consistently relevant throughout the study period. Recent advancements in artificial intelligence (AI) tools have further enhanced the application of digital spatial profiling in clinical trials.

## 4 Discussion

### 4.1 General situation

In this study, we constructed a comprehensive knowledge map of TIM-3 research in cancer. By analyzing diverse aspects, such as publication trends, geographical distribution, institutional partnerships, author influence, journal intersections, and thematic developments, valuable insights into TIM-3 in cancer research were obtained. These findings not only accurately reflect the current state of research but also play a crucial guiding role in informing future research directions, rational resource allocation, and the development of scientific policies. The novelty of this study lies in the amalgamation of multiple bibliometric analysis methods to comprehensively introduce TIM-3 research in cancer from multiple perspectives, thereby providing a foundation for future advancements in the field.

We collected articles exploring TIM-3 research in cancer from the WoSCC database from January 2000 to June 2025. We included and analyzed a total of 1,466 articles published in 346 journals. 10,079 authors from 1,989 institutions in 66 countries contributed these articles. In addition, 37 articles from the PubMed database were included and analyzed.

The analysis revealed steady growth in publications related to TIM-3 research in cancer, with a notable increase in relevant publications after 2016. This surge can be attributed to an article titled “PD-1 says goodbye, TIM-3 says hello” published by Diana Romero et al. in Nature Reviews Clinical Oncology (56), which drew the attention of many researchers to TIM-3 as an immune checkpoint. The publication count peaked at 176 in 2022 but has slightly declined since then. This decrease may be associated with advanced achievements in cancer immunotherapy using PD-1. In 2006, the FDA approved atezolizumab, an anti-PD-L1 antibody, for the treatment of non-small cell lung cancer and advanced urothelial carcinoma (57). Currently, clinical data show that TIM-3 monotherapy does not exhibit remarkable antitumor efficacy (58). However, according to data presented at the 2022 ASCO Annual Meeting from the Phase 1 AMBER study, the combination therapy of cobalolimab (anti-TIM-3 antibody) and dostarlimab (anti-PD-1 antibody) demonstrated preliminary anticancer activity and manageable safety in the treatment of advanced or metastatic melanoma (59). Blocking the TIM-3/Galectin-9 and PD-1/PD-L1 pathways with TIM-3 and PD-1 monoclonal antibodies has become a way to restore immune function and target antitumor immunotherapy (31). Recent animal studies have demonstrated that the antitumor efficacy of combined TIM-3 and PD-1 therapy surpasses that of monotherapy (31, 32). Despite a decrease in the number of recent publications owing to various factors, research on TIM-3 in cancer still holds significant potential for further exploration.

The analysis of international cooperation reveals that China, the United States, and Japan are at the forefront of studying TIM-3 in cancer. It is worth noting that the United States has a centrality score of 0.96, which is significantly higher than that of other countries, indicating its central position in this research field. China leads globally in publishing volume in this domain; Nonetheless, it has a comparatively low average citation count per paper, accompanied by a significant home bias. Research indicates that self-citations from China constitute up to 57.2%, far surpassing the 37.1% observed in the United States. This citation preference constrains the global visibility and influence of Chinese publications (60). A growing number of low-quality research articles is a significant factor contributing to China’s comparatively low citation rates for published papers. The evaluative pressures and incentive structures encountered by Chinese scholars frequently compel them to prioritize the volume of publications over their quality (61).

In terms of institutional cooperation, the key institutions were mainly from the United States and China. However, most institutions have lower centrality scores, suggesting that the majority of these institutions still have a low impact and that there is insufficient cooperation among them. This may also constrain the innovative pathways of various institutions, making it difficult for them to participate in groundbreaking interdisciplinary research. Primary factors include disparities in financing, equipment, and data between institutions, as well as potential conflicts in collaborations stemming from unequal resource allocation, which may influence the propensity to collaborate. Moreover, institutions across various areas encounter geographic distance, time zone disparities, and linguistic obstacles, which elevate the communication expenses of international collaboration and constrain the frequency and profundity of cross-regional cooperation. Meanwhile, institutions with many cultural origins exhibit variations in collaborative conceptions and management strategies. Based on this, government can optimize its financial structures and establish cooperative incentive systems. Consequently, improving the funding structure and implementing a joint incentive mechanism are essential. Led by the government or leading academic institutions, establish specialized interdisciplinary and international cooperation funds aimed at supporting collaborative projects across diverse institutions and disciplines, while dynamically modifying funding amounts based on the actual results of these projects and promoting sustained and stable partnerships among institutions. Furthermore, a data-sharing platform may be created to reduce the effects of geographical distance on collaboration (62). Regarding author collaboration, the top two authors with the highest publication volume were both from Harvard University in the United States, further highlighting the institution’s dominance in this domain. This also underscores America’s leading position, but it may lead to issues such as resource concentration and bias in disease areas. Moving forward, the global research landscape must pursue scientific progress through multipolar collaboration and inclusive policies, ensuring that research outcomes benefit all populations.

Co-occurrence and co-citation analyses revealed that the top ten authors contributed only 11.74% of the articles, with the most prolific author publishing fewer than 50. This suggests that, despite the involvement of numerous authors, the quantity of highly productive authors remains limited. Among them, Anderson AC, stands out as one of the top ten authors and co-cited authors, marking her as a key figure in this research field. Anderson AC et al. have made profound contributions to the study of TIM-3 in cancer research, particularly in advancing its role in cancer survival prognosis. They found that inhibiting TIM-3 in syngeneic models of diffuse pontine glioma can extend survival and produce disease-free patients with immunological memory (63). Journals such as The Journal of Immunology and Nature Immunology have become essential platforms for disseminating high-impact research, while journals such as Frontiers in Oncology and the International Journal of Molecular Sciences have gained increasing influence in recent years. These journals primarily aim to enhance our understanding of cancer epidemiology through various aspects, including molecular biology, and play a key role in cancer diagnosis and treatment.

We employed keyword co-occurrence analysis to exhibit thematic evolution and untangle the focal points and significant trends of TIM-3 research in cancer. In bibliometrics, network diagrams of keyword co-occurrences can reveal these themes (64). The clustering of co-occurring keywords indicated five distinct clusters in this field. The green cluster comprised 36 keywords. The most prominent nodes were TIM-3, T cells, response, receptor, pathway, expression, activation, and immunity. This cluster likely focuses on the role of the TIM-3 receptor, including its expression and activation in T cells, as well as its associated pathways and their impact on immunity and immune responses. The red cluster, characterized by the prominent keywords survival, lymphocyte, PD-L1, immunotherapy, and cancer (containing 38 keywords in total), primarily investigates the relationship between PD-L1, lymphocytes, the efficacy of immunotherapy, and patient survival in cancer research. The blue cluster contains studies that may explore the two immune checkpoint molecules PD-1 and LAG-3, as well as their associations with immune cell exhaustion and dysfunction. It includes 20 keywords related to blockade strategies for these two immune checkpoint molecules, including PD-1, LAG-3, exhaustion, dysfunction, and blockade. The yellow cluster contained 17 keywords, mainly LAG-3, up-regulation, antitumor immunity, CLTA-4, and NK cells, which may pertain to the examination of regulatory mechanisms of immunological checkpoints and the functional alterations of immune cells during the anti-tumor response. The purple cluster contained 7 keywords: inhibition, target, stem-cells, and acute myeloid leukemia, likely focusing on therapeutic approaches for leukemia. From the above analysis, it can be concluded that within the field of cancer research, immune checkpoints (TIM-3, PD-1, PD-L1, and LAG-3) and their associated pathways are linked to the expression, activation, exhaustion, and dysfunction of immune cells (T cells, NK cells, and lymphocytes) and stem cells. The blocking strategies targeting these immune checkpoints have an impact on the cancer immune response, tumor proliferation, and metastasis, as well as on patient survival and prognosis.

### 4.2 TIM-3 in cancer

#### 4.2.1 The immunosuppressive mechanism of TIM-3: multidimensional regulation of tumor immune evasion

**Figure 9:**
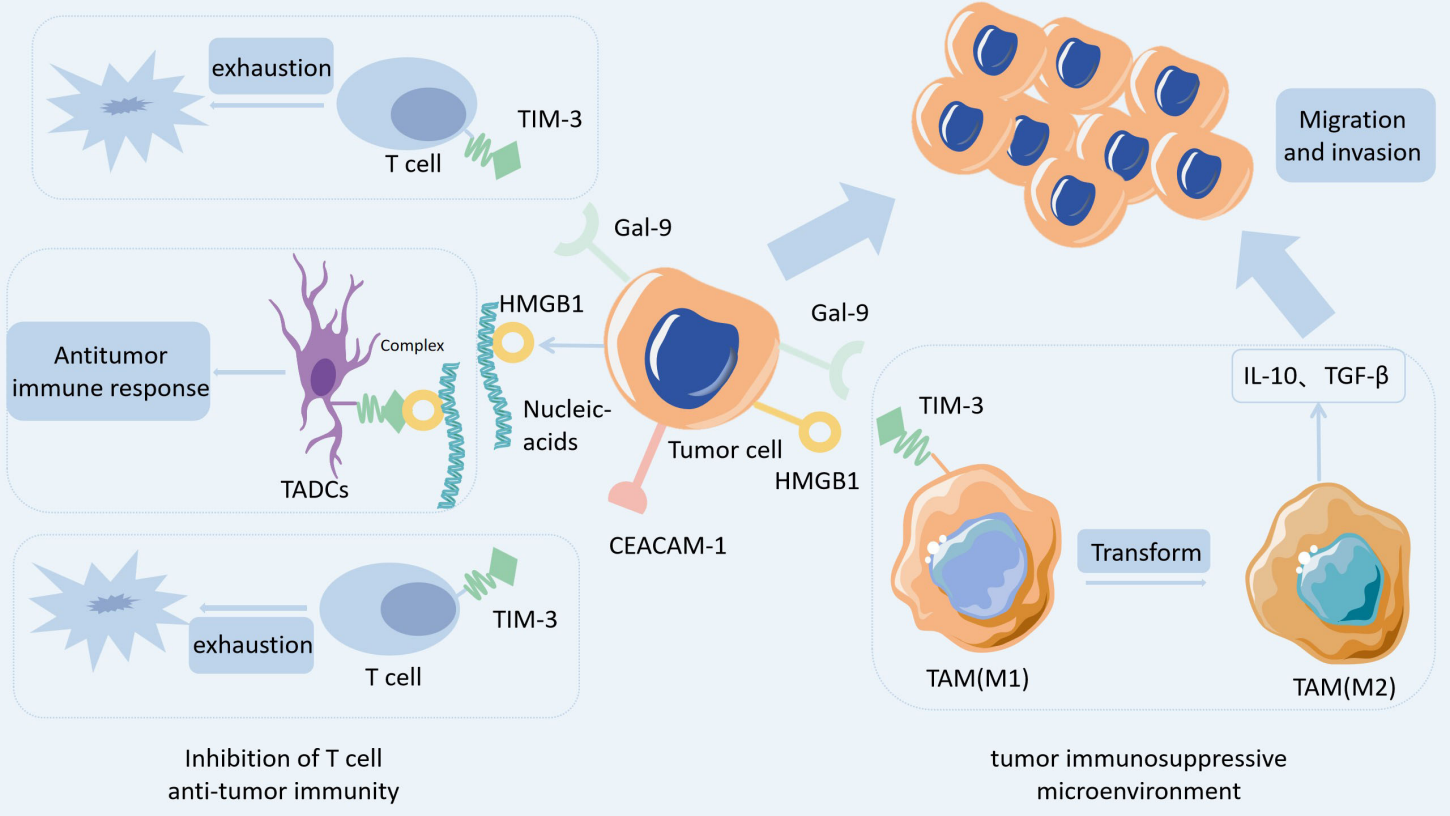
TIM-3 mediates multidimensional regulation of the tumor immune evasion map.

TIM-3, an important immune checkpoint molecule, plays a pivotal role in cancer development by regulating immune cell function and immunosuppressive pathways within the Tumor Microenvironment (TME) (**Figure 9**). TIM-3 is expressed on T cells (except for Th2 cells) and other immune cells, such as NK cells, macrophages, dendritic cells (DCs), myeloid-derived suppressor cells, and mast cells (65). In cancer patients, TIM-3 overexpression can be detected in most immune cells, particularly in antigen-specific CD8+ T cells, CD4+ T cells, and NK cells (66, 67). In melanoma, tumor antigen-specific CD8+ T cells among tumor-infiltrating lymphocytes (TILs) co-express TIM-3 and PD-1, leading to reduced secretion of cytokines such as interferon-gamma (IFN-γ) and tumor necrosis factor-alpha (TNF-α), as well as diminished proliferative capacity; this phenotype is closely related to disease progression (68, 69). Studies have revealed that TIM-3 can bind to its ligand Galectin-9, triggering calcium flux and caspase activation in Th1 cells, thereby inducing apoptosis of Th1 cells (28). It also forms heterodimers with Ceacam1, further exacerbating T cell exhaustion (70). Moreover, TIM-3 expression is upregulated on tumor-associated dendritic cells (TADCs), where it interacts with HMGB1 on the surface of tumor cells. This interaction suppresses nucleic acid-mediated anti-tumor immune responses and promotes cancer immune evasion (71). TIM-3 is predominantly expressed on tumor-infiltrating CD4+ and CD8+ T cells in non-small cell lung cancer (NSCLC). Among CD4+ TILs, the proportion of Foxp3+ regulatory T cells (Tregs) is significantly elevated in TIM-3+ CD4+ T cells, and their frequency is positively correlated with poor patient prognosis, suggesting that TIM-3 promotes tumor immune evasion by enhancing the immunosuppressive function of Tregs (72). Furthermore, TIM-3 is highly expressed on tumor-infiltrating NK cells (TINKs) in patients with esophageal cancer, and the levels of perforin and granzyme B are significantly reduced in TIM-3+ TINKs. TNF-α induces TIM-3 expression via the NF-κB pathway, leading to NK cell exhaustion. Higher TIM-3 expression in TINKs in esophageal cancer patients is associated with tumor invasion, lymph node status, and advanced stage, indicating inhibition of anti-tumor activity (73).

Furthermore, TIM-3 contributes to tumor immune evasion by remodeling the tumor microenvironment. It is expressed on myeloid cells, such as tumor-associated macrophages (TAMs), and interacts with ligands such as Ceacam1 or HMGB1, promoting the polarization of macrophages toward a pro-tumor phenotype (M2 type) and inhibiting their antigen-presenting capacity (74, 75). Concurrently, these immunosuppressive cells secrete inhibitory factors, including IL-10 and TGF-β, which further suppress T cell-mediated anti-tumor immune responses, thereby facilitating tumor growth and dissemination (76). Together, these mechanisms constitute the “immunosuppressive tumor microenvironment”.

#### 4.2.2 The application potential and development prospects of TIM-3 in cancer

##### 4.2.2.1 TIM-3 can serve as a prognostic marker for cancer

Numerous clinical studies have demonstrated that high TIM-3 expression is significantly associated with poor prognosis. Wang et al. found that Tim-3 is minimally expressed in gastric cancer cells, whereas its ligand Galectin-9 is markedly overexpressed in tumor cells. The expression levels of TIM-3 and Galectin-9, as well as the density of Foxp3+T cells, were negatively correlated with the overall survival (OS) rate of patients (67). Among patients with gastric cancer, those with lymphovascular invasion exhibited higher TIM-3 expression levels (*P* < 0.001). Both high Galectin-9 expression and low TIM-3 expression were significantly associated with prolonged overall survival (*P* = 0.002 and *P* = 0.010, respectively). The combined expression of Galectin-9 and TIM-3 served as independent prognostic predictors for patients with gastric cancer (RR: 0.43; 95% CI: 0.20–0.93). Thus, the expression of Galectin-9 and TIM-3 in tumor cells may represent potential independent prognostic factors for gastric cancer patients (77). Furthermore, a Phase I trial (NCT02573363) suggested that targeting the Galectin-9/TIM-3 axis in combination with induction chemotherapy may effectively increase the likelihood of complete remission in patients with acute myeloid leukemia (AML) (78).

##### 4.2.2.2 TIM-3 targeted therapy for cancer

Based on the immunomodulatory effects of TIM-3, it has become an important target for cancer immunotherapy. Current research focuses on monotherapy, combination strategies, and the development of novel therapies, which show promising potential for wide application in the future.

Anti-TIM-3 monoclonal antibody reverses immune cell exhaustion by blocking its interaction with ligands. In murine tumor models (such as MC38 colon cancer and B16 melanoma), inhibition of CD8+ T cells can be alleviated by blocking TIM-3, restoring their proliferative capacity and cytotoxic function. However, due to the complex pathophysiology of tumors, immunotherapy targeting a single antigen often fails to effectively eliminate target cells. Patients subjected to single-antibody therapy may develop treatment resistance or fail to respond. Monotherapy with anti-TIM-3 delays tumor growth, and in a TRAMP-C1 prostate cancer model, its combination with anti-PD-1 synergistically enhances the cytotoxicity of CD8+ T cells (79, 80). Considering the results of various preclinical studies investigating monoclonal antibodies that block TIM-3, along with findings from early-stage clinical trials that are still ongoing, TIM-3 immune checkpoint blockade (ICB) shows promise as a potential new approach to cancer immunotherapy, particularly when combined with other ICB agents. In a mouse model of lung cancer, after resistance to anti–PD-1 antibody treatment, TIM-3 expression was upregulated, and the combined blockade of TIM-3 prolonged mouse survival (median survival increased from 5 weeks to 11.9 weeks). In a glioblastoma model, dual blockade of TIM-3 and PD-1 increased the survival rate of mice from 27.8% to 57.9% (80). A clinical study (NCT03680508) demonstrated that the combination of TSR-022 (cobolimab, a TIM-3 binding antibody) and TSR-042 (dostarlimab, a PD-1 binding antibody) resulted in a significantly higher objective response rate in patients with hepatocellular carcinoma compared to monotherapy. TSR-022 and TSR-042 may stop the growth of tumor cells by allowing the immune system to attack the cancer (81). Bispecific antibodies (BsAbs) are artificially engineered antibodies produced through cell fusion, recombinant DNA technology, or protein engineering. They are capable of binding simultaneously or sequentially to two distinct antigens or two different epitopes on the same antigen (82). RO7121661 currently targets both TIM-3 and PD-1 simultaneously. By synergistically relieving immune suppression and enhancing T-cell infiltration, it has entered Phase I clinical trials (NCT03708328) for the treatment of advanced solid tumors. Furthermore, the combined blockade of TIM-3 with LAG-3 and TIGIT is also being explored in clinical trials (such as NCT04370704) to further alleviate tumor immune evasion (65).

#### 4.2.3 Existing issues and development prospects

Despite the promising prospects of TIM-3-targeted therapy, several issues remain unaddressed. First, the biological functions of TIM-3 are complex and diverse, and its non-canonical signaling pathways have not yet been fully elucidated. Therefore, the potential side effects and safety concerns associated with TIM-3 antibody-based drugs must be thoroughly considered during their development. Second, the expression level of TIM-3 may vary across different types of tumors and among individual patients, which could lead to the inconsistent efficacy of TIM-3-targeting antibodies. To address these challenges, researchers are actively seeking novel biomarkers to predict the response to TIM-3 antibody therapies and exploring combination strategies to optimize treatment plans.

To provide a basis for precision therapy, researchers need to further investigate the functional heterogeneity of TIM-3 across different cell types (such as T cells and myeloid cells) and elucidate its regulatory networks within various tumor microenvironments. It is also essential to explore the synergistic effects of TIM-3 with radiotherapy, chemotherapy, and targeted agents. For instance, radiotherapy can upregulate TIM-3 expression, and its combination with TIM-3 monoclonal antibodies may enhance immunogenic cell death (80). Through in-depth investigation of the structure and function of TIM-3, development of highly effective TIM-3 antibody drugs, and exploration of combination therapy strategies, we expect to provide novel insights and approaches for cancer treatment. With continuous technological advancements and further clinical research, TIM-3 antibodies are anticipated to play an even more profound role in oncology therapeutics, offering hope to more patients.

#### 4.2.4 Strengths and limitations

To gain a thorough insight into the present state of TIM-3 research, this study employed bibliometrics and related software, including CiteSpace and VOSviewer, to elaborate on the research journey in this field, explore current hotspots and prospects, and identify collaborative relationships among authors, countries, and institutions. However, considering the various factors, this study has certain limitations. First, data were retrieved only from WoSCC and PubMed, covering publications in SSCI and SCIE. The publication timeframe was limited to 2000-2025, resulting in a total of 1,466 articles and 37 clinical trials retrieved. Although the PubMed database has been added, databases such as Scopus, which are reputable search engines, have not been included in the study. Additionally, since TIM-3 was first discovered in 2002 (21), articles published before 2000 were excluded from this study.

Second, English is a universal language, and all the included literature is in English. We did not include relevant literature in other languages, which may have resulted in a language bias (83). Such a situation might result in an insufficient depiction of international research trends; thus, our findings may not apply to studies published in other languages. Furthermore, bibliometric analysis requires a certain period of data collection and statistical analysis. Therefore, the analysis conducted before updating citation data may not reflect the latest research impact, and recently published articles may also have a strong influence. The effectiveness of clinical treatment programs or medications frequently requires validation through empirical research, such as clinical trials. Bibliometrics, however, focuses on the assessment of publications and citation metrics and is incapable of directly evaluating treatment outcomes.

## 5 Conclusion

Recently, research on TIM-3 immunotherapy in cancer has garnered increasing attention. This is evidenced by the surge in annual publications since 2003. This article identified the top global researchers and institutions involved in the study of TIM-3 in cancer. Frontiers in Immunology is the most active journal in this field, and Anderson AC is the core author. Furthermore, 37 clinical trial articles sourced from the PubMed database were also included. The United States is a pioneer in clinical research on TIM-3 in the field of cancer. Among the authors, Leena Gandhi published the highest number of articles. In cancer research, the immune checkpoint TIM-3 and its related pathways are associated with the expression, activation, exhaustion, and dysfunction of immune cells (such as T cells and natural killer cells) and stem cells. Blockade strategies targeting these immune checkpoints influence the cancer immune response, tumor proliferation, and metastasis, which represents a current research hotspot. The development of combination strategies and novel therapies may be key areas for future investigation.

## Data Availability

The original contributions presented in the study are included in the article/**Supplementary Material**. Further inquiries can be directed to the corresponding author.
