# Correction: Bibliometric analysis of knowledge maps and future trends of TIM-3 in cancer

**DOI:** 10.3389/fonc.2026.1823035

**Published:** 2026-04-13

**Authors:** Shuchi Lv, Shanchuan Huang, Langming Li, Kaifeng Lin, Hongliang Zhang, Yuqi Huang

**Affiliations:** 1 Department of Orthopaedics, Dongguan Humen Hospital, Dongguan, China; 2 Department of Breast Center, Dongguan Kanghua Hospital, Dongguan, China

**Keywords:** bibliometric, cancer, CiteSpace, immunotherapy, TIM-3, VOSviewer

Affiliation ^1^Department of Orthopaedics, Dongguan Humen Hospital, Dongguan, China

was erroneously given as ^2^Department of Orthopaedics, Dongguan Humen Hospital, Dongguan, China.

Affiliation ^2^Department of Breast Center, Dongguan Kanghua Hospital, Dongguan, China

was erroneously given as ^1^Department of Breast Center, Dongguan Kanghua Hospital, Dongguan, China.

The original version of this article has been updated.

