# Supplementary material for: Bibliometric analysis of knowledge maps and future trends of TIM-3 in cancer
Source: Front Oncol. 2026 Feb 10;16:1766463. doi: 10.3389/fonc.2026.1766463 (PMC12930370; doi:10.3389/fonc.2026.1766463)
Supplement: Supplementary file 1 [file DataSheet1.docx]

Supplementary Materials of the article.

# Supplementary Figures and Tables

## Supplementary Figures


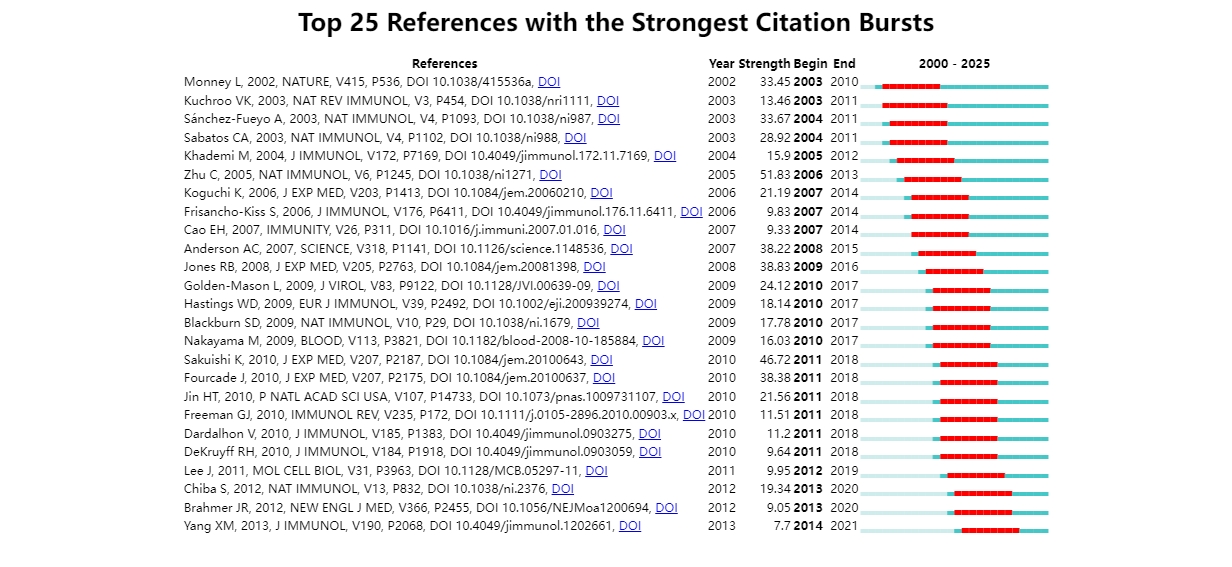


**Supplementary Figure 1.** Top 25 highly cited references with citation bursts in the field of cancer research on TIM-3.

## Supplementary Tables

**Supplementary Table 1** The keywords burst with a burst period from the beginning to 2025.

| Begin | **End** | Strength | Year | **Entity** |
| --- | --- | --- | --- | --- |
| 2020 | 2025 | 3.47 | 2020 | tumors |
| 2021 | 2025 | 7.97 | 2018 | immune checkpoint inhibitors |
| 2021 | 2025 | 5.58 | 2018 | pd l1 |
| 2021 | 2025 | 4.68 | 2018 | open label |
| 2021 | 2025 | 4.21 | 2019 | combination |
| 2021 | 2023 | 3.61 | 2006 | immune response |
| 2022 | 2025 | 4.56 | 2019 | microenvironment |
| 2022 | 2025 | 3.96 | 2022 | multiple myeloma |
| 2020 | 2025 | 4.85 | 2020 | stem cells |
| 2023 | 2025 | 8.09 | 2017 | tumor microenvironment |
| 2023 | 2025 | 5.28 | 2003 | cells |
| 2023 | 2025 | 4.38 | 2019 | risk |
| 2023 | 2025 | 3.99 | 2007 | cancer |
| 2023 | 2025 | 3.22 | 2018 | efficacy |
| 2024 | 2025 | 4.17 | 2024 | mutations |

**Supplementary Table 2** The most trafficked keyword for the top five modules each year.

| **Year** | | **2003** | | **2004** | | **2005** | | **2006** | | **2007** | | **2008** | | **2009** | |
| --- | --- | --- | --- | --- | --- | --- | --- | --- | --- | --- | --- | --- | --- | --- | --- |
| Total modules | | 2 | | 6 | | 4 | | 8 | | 7 | | 6 | | 8 | |
| Module1 | | Gene family | | Th cell | | Mice | | Immune deviation | | Murine model | | Cutting edge | | Epidermal hyperplasia | |
| Module2 | | Donor-specific transfusion | | subsets | | Kidney injury | | B7-h1 | | proliferation | | Hepatitis A virus | | DNA | |
| Module3 | | - | | immunity | | Peripheral tolerance | | Helper type 1 responses | | Gene family | | expression | | expansion | |
| Module4 | | - | | responses | | gene | | cytokine | | Peripheral tolerance | | family | | Epithelial cells | |
| Module5 | | - | | identification | | - | | immune response | | pathway | | Factor alpha | | Dying cells | |
| **Year** | **2010** | | **2011** | | **2012** | | **2013** | | **2014** | | **2015** | | **2016** | | **2017** |
| Total modules | 8 | | 7 | | 13 | | 9 | | 9 | | 9 | | 9 | | 12 |
| Module1 | blockade | | family | | Chemokine production | | Tim 3 ligand | | Anti-CTLA-4 | | Transcription factor | | Tumor-infiltrating lymphocytes | | Ovarian cancer |
| Module2 | distinct | | Autoimmune disease | | Atopic dermatitis | | "b7 family | | Therapeutic target | | Activated T cells | | pathway | | non-small_cell_lung_cancer |
| Module3 | Dc hil | | binding | | cd4(+) t cells | | Alternative activation | | inflammation | | vaccination | | Adenosine receptor | | In vivo |
| Module4 | Breast cancer | | Containing molecule 3 tim 3 | | Bone marrow transplantation | | tolerance | | Multiple sclerosis | | survival | | t-cell exhaustion | | persistence |
| Module5 | Chinese han population | | Bone marrow transplantation | | Acute myeloid leukemia | | Autoimmune disease | | Anti-PD1 antibody | | A vaccine | | Virus infection | | phosphorylation |

| **Year** | **2018** | **2019** | **2020** | **2021** | **2022** | **2023** | **2024** | **2025** |
| --- | --- | --- | --- | --- | --- | --- | --- | --- |
| Total modules | 13 | 17 | 14 | 18 | 20 | 16 | 17 | 6 |
| Module1 | macrophages | marker | Inhibitory receptors | conversion | Cutting edge | Advanced solid tumors | Hodgkin lymphoma | Nk cells |
| Module2 | Natural killer cells | biomarker | in vivo | generation | Ifn gamma | diagnosis | Up regulation | Microsatellite instability |
| Module3 | multicenter | diagnosis | Liver cancer | evolution | Solid tumors | Tumor-infiltrating lymphocytes | Immune cells | Myeloid-derived suppressor cells |
| Module4 | leukemia | memory | foxp3 expression | Antitumor activity | Disease progression | Receptor tim-3 | Hematological malignancies | inhibition |
| Module5 | effector | Ctla-4 blockade | Anti pd_1 | Flow cytometry | Hepatocellular carcinoma | Gastroesophageal junction | Immune checkpoint inhibitor | Immune resistance |

**Supplementary Table 3 Top ten most productive authors in clinical trials related to TIM-3 in cancer research.**

| **Rank** | **Author** | **Documents(n%)** | **Conutry** |
| --- | --- | --- | --- |
| 1 | Leena, Gandhi | 3（8.11%） | USA |
| 2 | M, Szpurka Anna | 3（8.11%） | USA |
| 3 | Stephen, Hodi F | 3（8.11%） | USA |
| 4 | Toshihiko, Doi | 3（8.11%） | Japan |
| 5 | B Lesinski Gregory | 3（8.11%） | USA |
| 6 | A Braun David | 2（5.41%） | USA |
| 7 | A, Shukla Sachet | 2（5.41%） | USA |
| 8 | Abdallah, Flaifel | 2（5.41%） | USA |
| 9 | B, Atkins Michael | 2（5.41%） | USA |
| 10 | Boris, Calderon | 2（5.41%） | USA |
